# Influence of Polymer Concentration on Drying of SPION Dispersions by Electrospinning

**DOI:** 10.3390/pharmaceutics15061619

**Published:** 2023-05-30

**Authors:** Črt Dragar, Žan Rekar, Tanja Potrč, Sebastjan Nemec, Slavko Kralj, Petra Kocbek

**Affiliations:** 1 Department of Pharmaceutical Technology, Faculty of Pharmacy, University of Ljubljana, SI-1000 Ljubljana, Slovenia; crt.dragar@ffa.uni-lj.si (Č.D.); tanja.potrc@ffa.uni-lj.si (T.P.); sebastjan.nemec@ijs.si (S.N.); slavko.kralj@ijs.si (S.K.); 2 Department for Materials Synthesis, Jožef Stefan Institute, SI-1000 Ljubljana, Slovenia; 3 Nanos SCI, Nanos Scientificae d.o.o., SI-1000 Ljubljana, Slovenia

**Keywords:** drying, electrospinning, nanofibers, poloxamer 188, polyethylene oxide, superparamagnetic iron oxide nanoparticles

## Abstract

To improve the physical stability of nanoparticle dispersions, several methods for their transformation into stable and easily dispersible dry products have been investigated thus far. Recently, electrospinning was shown to be a novel nanoparticle dispersion drying method, which addresses the crucial challenges of the current drying methods. It is a relatively simple method, but it is affected by various ambient, process, and dispersion parameters, which impact the properties of the electrospun product. The aim of this study was, thus, to investigate the influence of the most important dispersion parameter, namely the total polymer concentration, on the drying method efficiency and the properties of the electrospun product. The formulation was based on a mixture of hydrophilic polymers poloxamer 188 and polyethylene oxide in the weight ratio of 1:1, which is acceptable for potential parenteral application. We showed that the total polymer concentration of prior-drying samples is closely related to their viscosity and conductivity, also affecting the morphology of the electrospun product. However, the change in morphology of the electrospun product does not affect the efficiency of SPION reconstitution from the electrospun product. Regardless of the morphology, the electrospun product is not in powder form and is therefore safer to handle compared to powder nanoformulations. The optimal total polymer concentration in the prior-drying SPION dispersion, which enables the formation of an easily dispersible electrospun product with high SPION-loading (65% (*w*/*w*)) and fibrillar morphology, was shown to be 4.2% (*w*/*v*).

## 1. Introduction

Since the approval of the first nanotechnology-based drug delivery system by the Food and Drug Administration in the early 2000s, nanotechnological approaches have gained significant attention in biomedicine [1,2,3]. Among several nanostructures which have been investigated until today, nanoparticles are the most important and promising for biomedical applications [4,5]. Typically, nanoparticles are in dispersion form and are therefore prone to chemical, microbiological, and physical instabilities [6]. Although suitable excipients, such as antioxidants and preservatives, can improve chemical and microbiological stability [6,7], the physical instability of nanoparticle dispersions remains an important technological challenge [8]. Among several approaches which have been investigated to improve the physical stability of nanoparticle dispersions, the transformation of the nanoparticle dispersion into a dry product was shown to be one of the most promising, since it does not affect the initial properties of the nanoparticles [8].

In the last two decades, several methods have been developed and investigated to transform nanoparticle dispersion into a stable and easily dispersible dry product. These drying methods include freeze-drying [6,7,9,10,11], spray-drying [12,13,14,15,16,17,18,19], spray–freeze drying [20,21,22], conventional drying in a heated oven [23,24], alcohol desiccation [23,25], vacuum drying [24], fluid-bed granulation [12,26,27,28], fluid-bed pellet coating [29], and supercritical fluids drying [30]. However, the majority of these drying methods has certain important limitations, which hinder their broader use in the drying of nanoparticle dispersions. Some of these limitations are (i) the introduction of the stress factors that may cause nanoparticle aggregation; (ii) relatively high amounts of additional excipients required; and (iii) the fine powdered form of the dry product, which raises safety concerns of having nanoparticle dust in the air during manipulation.

Recently, electrospinning emerged as a novel and promising method for drying nanoparticle dispersions by incorporating them into polymeric nanofibers. It preserves the initial properties of nanoparticles and enables their simple and rapid reconstitution [31]. The electrospinning, which has been widely used for nanofiber preparation, is based on the generation of a strong electric field between the conductive screen, i.e., the nanofiber collector, and the metal needle on the syringe containing a polymer solution or a polymer melt [32]. The polymer solution or melt is slowly squeezed out of the syringe, and the applied electrical voltage causes the cone-shaped deformation of the liquid droplet, known as the Taylor cone, which is followed by the ejection of a charged liquid jet stream. The electrically charged viscoelastic jet travels first in a straight line and then starts to bend under the influence of an electric field, generated between a grounded collector and a metal needle [33]. The viscoelastic jet elongates, the solvent rapidly evaporates (or the polymer melt cools), and continuous solid nanofibers are deposited on a grounded collector [34,35]. The physics of electrospinning is extremely complex, but the process itself is relatively simple and enables the production of a dry nanomaterial with controllable properties [35]. However, to produce a dry nanomaterial with the desired properties, several parameters need to be controlled, namely the ambient conditions, the process parameters, and the properties of solution/dispersion to be electrospun [35,36]. The temperature and relative humidity of the environment are usually considered to be ambient parameters [35,36,37]. Applied voltage, nozzle/needle-to-collector distance, a flow rate of the electrospinning solution/dispersion, nozzle and/or needle (diameter and design), and collector (composition, geometry, and rotation speed) are the most important process parameters and, thus, should be carefully controlled to obtain the electrospun product with the desired properties [35,36,37,38]. The dispersion parameters, namely the properties of selected polymer (type, molecular weight, and concentration), viscosity, conductivity, surface tension, and type of solvent (polymer solubility, permittivity, and solvent vapor pressure or boiling point), importantly influence the outcome, as well the properties of the final electrospun product [35,36,37,38]. Although the most important parameters that affect the electrospinning process and the properties of the obtained product are well-known, the prediction of the electrospinning outcome is very challenging due to the interplay of several known and probably some still unknown parameters [35,38]. The impact of the parameter variations is therefore not always straightforward since it also depends, amongst other things, on a polymer–solvent combination [35]. Among the dispersion parameters, the polymer concentration was shown to be the one that influences the electrospinning process most significantly and has an important impact on the viscosity of the polymer solution [38].

Until today, nanoparticles have demonstrated remarkable potential for their use in biomedicine, with iron-oxide-based magnetic nanoparticles being one of the most promising in recent years [3,5,39]. These nanoparticles possess unique physical, chemical, and biological properties, as well as superparamagnetic properties at sizes smaller than approximately 15 nm. They are used for nuclear magnetic resonance imaging as negative contrast agents, for magnetic hyperthermia in the treatment of recurrent glioblastoma multiforme, and for the treatment of iron-deficiency anemia [3,39]. Superparamagnetic iron oxide nanoparticles (SPIONs) can be guided in vivo by an external magnetic field gradient and generate heat in vivo when exposed to an alternating magnetic field at high frequencies. With an additional possibility to detect them in vivo with nuclear magnetic resonance imaging, SPIONs have great potential for their use in a wide range of biomedical applications, such as targeted drug delivery, tissue engineering, magnetofection, theranostics, and cell therapy [3,40,41,42]. However, the physical stability of SPION dispersions remains one of the crucial technological challenges that needs to be addressed to enable the successful translation of SPION-based nanoformulations from research to clinical practice [31].

Recently, electrospinning has been shown to address the crucial shortcomings of the currently available nanoparticle dispersion drying methods [31]. However, comprehensive systematic studies are needed to consolidate it as an efficient method for drying nanoparticle dispersions and to investigate the influence of the most important parameters on the drying process, as well as on the properties of the dry product. Thus, the aim of our present study was to evaluate the impact of the dispersion parameters on the drying efficiency and the properties of the obtained electrospun products, as shown in the schematic workflow of the overall work (Supplementary Materials Figure S1). The water-based dispersions of citrate-coated SPIONs were used as a challenging formulation to be dried and reconstituted efficiently.

## 2. Materials and Methods

### 2.1. Materials

All materials used were of reagent grade and from commercial sources. Iron (III) sulphate hydrate, iron (II) sulphate heptahydrate (ACS, 99+%), and citric acid (99+%) were purchased from Alfa Aesar (Haverhill, MA, USA). Acetone (AppliChem GmbH, Darmstadt, Germany), ethanol absolute (Carlo Erba Reagents GmbH, Emmendingen, Germany), NH_4_OH (aq) 25% (Honeywell Fluka, Charlotte, NC, USA), and HCl 1 M (Honeywell Riedl-de-Haën, Charlotte, NC, USA) were used as received. Polyethylene oxide (PEO; Mw, 400,000 g/mol) was from Sigma-Aldrich, Co. (St. Louis, MO, USA), and poloxamer 188 (P188; Lutrol F68) was from BASF (Ludwigshafen, Germany). The water used was purified by reverse osmosis.

### 2.2. Preparation of the Initial SPION Dispersion

The maghemite (γ-Fe_2_O_3_)-based SPIONs were produced by the precipitation method described in our previous publications [43,44]. In brief, an aqueous solution of Fe^2+^ (0.027 mol/L) and Fe^3+^ (0.023 mol/L) ions was precipitated with concentrated ammonia in two steps. In the first step, the pH value of the solution was set to pH 3 and maintained at this value for 30 min to precipitate the iron hydroxides. In the second step, the pH value was increased to a pH of 11.6 to oxidize the iron (II) hydroxide with air oxygen, forming a spinel product. After an aging of 30 min, the produced nanoparticles, namely SPIONs, were thoroughly washed with a diluted ammonia solution at a pH of 10.5.

The SPIONs were used for the preparation of the water-based initial SPION dispersion, i.e., ferrofluid [45,46]. A total of 2.5 mL of an aqueous solution of citric acid (0.5 g/mL) was added to the dispersion of washed SPIONs (approximately 1 g of SPIONs in 30 mL of water) while being rigorously stirred. The pH value was set to 5.2 with an ammonia solution and heated at 80 °C for 90 min in an oil bath equipped with a water condenser. Finally, the citrate-coated SPIONs were sedimented using a NdFeB magnet (Q-60-30-15-N, Supermagnete, Gottmadingen, Germany), washed twice with 100 mL of acetone, and dispersed in 30 mL of purified water. The aqueous SPION suspension was centrifuged for 10 min at 7500× *g* to remove any agglomerates of SPIONs. The obtained SPION dispersion with a SPION concentration of 560 mg/mL was stored at 8 °C. In this manuscript, it is referred to as the initial SPION dispersion.

### 2.3. Characterization of SPIONs in the Initial Dispersion

The initial dispersion was characterized regarding the SPION hydrodynamic size and their surface charge, using photon correlation spectroscopy (Zetasizer Ultra, Malvern Panalytical Ltd.; Worcestershire, UK) and laser Doppler electrophoresis (Zetasizer Ultra, Malvern Panalytical Ltd.; Worcestershire, UK), respectively. Before the measurements, the initial SPION dispersion was diluted with purified water to the final SPION concentration of 0.1 mg/mL, as recommended by the producer of Zetasizer Ultra (Malvern Panalytical Ltd.), and measured in triplicate. The results are expressed as average hydrodynamic size and average zeta potential, with corresponding standard deviations.

The size, morphology, and internal structure of the SPIONs were examined by transmission electron microscopy (TEM; Jem 2100; Jeol, Akishima, Japan). A drop of the initial SPION dispersion was placed on a carbon-coated copper TEM grid and air-dried at room temperature. TEM imaging was performed using an accelerating voltage of 200 kV. The mean SPION size was determined by measuring the diameter of at least one hundred randomly selected SPIONs on several representative TEM images, using ImageJ 1.53e software (National Institutes of Health; Bethesda, MD, USA).

### 2.4. Drying of SPION Dispersions

#### 2.4.1. Preparation of Prior-Drying Samples

To prepare the prior-drying samples (Table 1), the polymers, namely PEO and P188 in a weight ratio of 1:1, were firstly dissolved in purified water at 80 °C by moderate magnetic stirring. Next, the polymer solution was cooled to room temperature, and the required amount of the initial SPION dispersion was added to obtain the target concentration of SPIONs in the prior-drying dispersion (Table 1). The sample was mixed well to obtain a homogenous prior-drying dispersion, which was electrospun immediately after the preparation (see Section 2.4.2), resulting in a dry electrospun product (Table 1).

The prior-drying polymer solutions for the preparation of SPION-free electrospun products (formulations A_0_, B_0_, and C_0_; Table 1) were prepared in the same manner as prior-drying SPION dispersions (formulations A, B, and C; Table 1) without the addition of the initial SPION dispersion.

#### 2.4.2. Electrospinning

To prepare the electrospun products, the prior-drying samples (SPION dispersions or pure polymer solutions), which were prepared just before the electrospinning, were transferred to a 5 mL plastic syringe (Chirana, Stará Turá, Slovakia), which was placed in a syringe pump of the Spinbox Systems^®^ electrospinning device in the horizontal setting (Bioinicia, Valencia, Spain). The metal needle (Bioinicia, Valencia, Spain; outer diameter, 0.7 mm) was connected to the high-voltage generator and mounted on a plastic holder inside the electrospinning chamber, 15 cm away from the grounded collector, which was wrapped with an aluminum foil. The plastic holder with the metal needle and the syringe were connected using the plastic tube (outer diameter, 1.3 mm). The electrospinning process was performed for ~1 h at room temperature and relative humidity ≤ 45%, using a flow rate of 1.77 mL/h and a high voltage output of 15 kV. The obtained electrospun products were stored in a desiccator until further use to assure comparable moisture contents in all investigated samples.

#### 2.4.3. Oven-Drying and Lyophilization

The prior-drying SPION dispersion (formulation B) was dried in a heated oven and lyophilized as follows. An aliquot of SPION dispersion containing ~10 mg of the dry content was placed in a 20 mL glass vial and dried in a heated oven at 90 °C for ~1 h (FN 500, Nüve, Ankara, Turkey). Additionally, the initial SPION dispersion (11.6 µL) was also dried using the same procedure, resulting in ~6.5 mg of dry product (FN 500, Nüve, Ankara, Turkey). The oven-drying was performed in triplicates.

For lyophilization, 4 mL of prior-drying SPION dispersion was placed into a 20 mL glass vial and was frozen at −80 °C in a freezer. The lyophilization was performed in the Christ freeze-dryer (Martin Christ Gefriertrocknungsanlagen GmbH, Osterode am Harz, Germany). The primary drying was performed at a shelf temperature of −5 °C and pressure of 0.63 mbar for 24 h, followed by the secondary drying at a shelf temperature of 20 °C for 1 h.

### 2.5. Characterization of the Prior-Drying Samples

Prior-drying samples were characterized regarding their rheological properties, conductivity, and physical stability, as follows. The rheological properties of the prepared prior-drying samples were evaluated by a Physica MCR 301 rheometer (Anton Paar; Graz, Austria) with a cone-plate measuring system CP50-2 (cone diameter, 49.961 mm; cone angle, 2.001°; sample thickness, 0.209 mm). The rotational test with a controlled shear rate was performed at 25 ± 0.1 °C to measure the viscosity of the prior-drying samples, and the shear rate varied from 1 s^−1^ to 100 s^−1^. The oscillatory frequency sweep test was performed at a standard strain amplitude within the linear range (amplitude 1%) and temperature of 25 °C to evaluate the elastic and plastic modulus of prior-drying samples. The angular frequency during the oscillatory frequency sweep test varied from 0.1 rad/s to 100 rad/s.

The conductivity of the prior-drying samples was evaluated with an MC226 Conductivity Meter equipped with an Inlab^®^ 741 electrode (Mettler-Toledo; Greifensee, Switzerland). The measurements were performed in triplicates, and the results are expressed as average conductivities with corresponding standard deviations.

To evaluate the physical stability of the prior-drying SPION dispersions (formulations A, B, and C), the hydrodynamic size and surface charge of SPIONs in these dispersions were measured at the beginning and the end of the electrospinning process. The preparation of the samples and the analyses were conducted as described in Section 2.3.

### 2.6. Characterization of Electrospun Products

#### 2.6.1. Scanning Electron Microscopy

The morphology of the electrospun products was evaluated by scanning electron microscopy (SEM; Supra35 VP, Carl Zeiss; Oberkochen, Germany). The samples were attached to metal studs with double-sided conductive tape (diameter, 12 mm; Oxford Instruments; Oxon, UK), and imaging was performed using an accelerating voltage of 1 kV and a secondary electron detector. At least 100 measurements of nanofibers were performed based on several representative SEM images, using the ImageJ 1.53e software (National Institutes of Health; Bethesda, MD, USA), and the average nanofiber diameter with the corresponding standard deviation was calculated.

#### 2.6.2. Transmission Electron Microscopy

A carbon-coated copper TEM grid was attached to the grounded collector with double-sided conductive tape, and the samples were electrospun onto the grid for 2 min, as described in Section 2.4.2, to form a thin layer of the electrospun product. The internal structure of the electrospun products with SPIONs was then examined by transmission electron microscopy (TEM; Jem 2100; Jeol, Akishima, Japan) operated at 200 kV.

#### 2.6.3. Thermogravimetric Analysis

To determine the SPION content in the electrospun products, a sample (2–5 mg) was weighed in an aluminum oxide crucible (70 µL), and an analysis was performed in an inert atmosphere (nitrogen flow 50 mL/min) in a temperature range from 30 °C to 650 °C (10 °C/min), using a thermogravimeter TGA/DSC 1 STARe System (Mettler-Toledo; Greifensee, Switzerland). The SPION content in the sample was calculated using Equation (1):

(1) $$\mathrm{SPION}\;\mathrm{content}\;=\frac{m_{0}-m_{\mathrm{LOSS}}}{m_{0}\;}\times 100\%$$

where m_0_ is the initial mass of the electrospun sample, and m_LOSS_ is the mass loss in the temperature range from 200 to 450 °C, estimated by the TGA. The electrospun products were sampled from three different locations on the collector. The results are expressed as the average SPION contents, with the corresponding standard deviations. All the experiments were performed in triplicates.

The residual moisture in the electrospun product was determined based on the mass loss in the temperature range from 30 to 150 °C in the same TGA. All the results are expressed as an average with the corresponding standard deviation.

#### 2.6.4. FTIR Analysis

The electrospun products were analyzed with an FTIR spectrometer with an attenuated total reflectance accessory (Nexus; Thermo Nicolet, Madison, WI, USA). Spectra in the range of 600–3900 cm^−1^, with a resolution of 2 cm^−1^, were recorded, and each recorded spectrum was an average of 64 scans. Additionally, the pure polymers in powder form, namely PEO and P188, and SPIONs were analyzed individually and in the physical mixture, which was prepared in a mortar by hand mixing of 65 mg of SPIONs, 17.5 mg of PEO, and 17.5 mg of P188.

#### 2.6.5. Vibrating-Sample Magnetometry

The magnetization (*M*) of SPIONs and the electrospun product of formulation B was determined at an external magnetic field of 1 T, using a vibrating-sample magnetometer (VSM 7307, Lake Shore Cryotronics, Westerville, OH, USA). Before the measurement, the initial SPION dispersion was dried at 80 °C overnight. The electrospun product was not additionally treated before the magnetic measurements. The SPION content in the electrospun product was calculated using Equation (2):

(2) $$\mathrm{SPION}\;\mathrm{content}\;=\frac{M_{\mathrm{PRODUCT}}}{M_{\mathrm{SPIONs}}\;}\times 100\%$$

where M_PRODUCT_ is the magnetization of the electrospun product, and M_SPIONs_ the magnetization of SPIONs, both estimated by vibrating-sample magnetometry (VSM).

### 2.7. Reconstitution of SPIONs

To reconstitute the SPIONs from the electrospun products and reference dry products (i.e., oven-dried and lyophilized products), 10 mL of purified water was added to the sample (~10 mg) in a 20 mL glass vial and mixed well by 3 min vortex mixing. The dispersion was visually inspected to confirm the reconstitution. Next, the hydrodynamic size and zeta potential of SPIONs were evaluated, as described in Section 2.3. The short-term physical stability of the SPION dispersions after reconstitution was evaluated by measuring the hydrodynamic size and zeta potential of SPIONs every 30 min in the first hour after the reconstitution. The pH value of the reconstituted SPION dispersions was measured with the pH meter SevenCompact^TM^ pH/Ion S220 equipped with InLab^®^ Expert Pro-ISM electrode (Mettler-Toledo; Greifensee, Switzerland). All reconstitution experiments were performed in triplicates, and the results are expressed as an average with the corresponding standard deviation.

Additionally, a drop of the reconstituted SPION dispersion was placed on a carbon-coated copper TEM grid, air-dried at room temperature, and evaluated by transmission electron microscopy (TEM; Jem 2100; Jeol, Akishima, Japan). TEM imaging was performed using an accelerating voltage of 200 kV.

#### Evaluation of the Polymer Impact on the Hydrodynamic Size and Zeta Potential of SPIONs

To investigate the polymer impact on the SPION hydrodynamic size and zeta potential after the reconstitution in the purified water, a polymer solution with the addition of SPIONs was prepared. First, polymers, namely PEO and P188 in a weight ratio of 1:1, were dissolved in 10 mL of purified water at 80 °C by magnetic stirring to obtain a 0.035% (*w*/*v*) polymer solution, which was subsequently cooled to room temperature. Next, 11.6 µL of the initial SPION dispersion, containing 6.5 mg of SPIONs, was added to the polymer solution and mixed well to obtain a 0.065% (*w*/*v*) SPION dispersion in the 0.035% (*w*/*v*) polymer solution. The composition of the prepared SPION dispersion was similar to the sample obtained by the reconstitution of the electrospun product (see Section 2.7). The described dispersion was prepared in triplicate, and the hydrodynamic size and zeta potential of SPIONs were evaluated as described in Section 2.3.

### 2.8. Statistical Analysis

The data are expressed as average +/− standard deviation. The statistical analysis for the comparison of samples was performed with a one-way analysis of variance (ANOVA) with Tukey’s post hoc tests for multiple sample comparison or Student’s *t*-test for two-sample comparison, using OriginPro 2018 software (OriginLab Corporation, Northampton, MA, USA). Significance was tested at the 0.05 level of probability.

## 3. Results and Discussion

### 3.1. Characterization of SPIONs in the Initial Dispersion

**Figure 1:**
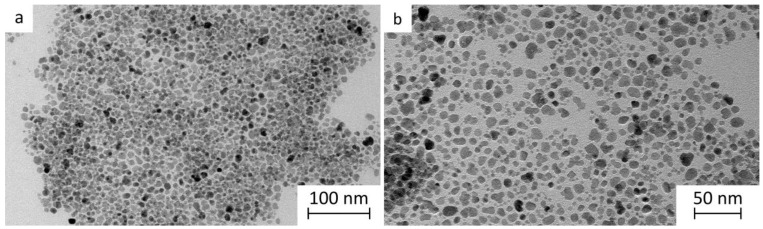
Representative TEM images of SPIONs in the initial SPION dispersion at (**a**) lower and (**b**) higher magnification.

The mean SPION size determined based on representative TEM images was (11.2 ± 2.2) nm (Figure 1). Their hydrodynamic size in purified water, determined with photon correlation spectroscopy (also known as dynamic light scattering [47]), was bigger, i.e., (53.5 ± 6.5) nm. This difference in the determined particle size of SPIONs is due to the difference in the measuring approach; namely, the hydrodynamic size of SPIONs is measured in dispersion, while TEM analysis is performed on dry SPION sample in vacuum and provides the size of individual nanocrystals. Thus, the hydrodynamic size also includes the solvated layer on SPIONs or its aggregates and is therefore commonly larger [47]. The SPIONs in the water-based initial dispersion show a single spinel phase and are maghemite, as evaluated in our previous study by X-ray powder diffraction and by Mossbauer microscopy [48,49].

The surface charge of SPIONs dispersed in purified water, characterized as the measurement of the zeta potential, was determined to be (−32.6 ± 7.7) mV. The zeta potential of nanoparticles importantly affects the physical stability of electrostatically stabilized nanoparticle dispersions, which can be classified as physically stable when the zeta potential is >±30 mV [50]. Thus, our initial SPION dispersion can be classified as physically stable.

### 3.2. Drying of SPION Dispersions

#### 3.2.1. Selection of the Composition of Prior-Drying Dispersions

To enable the formation of the electrospun product with the desired nanofibrillar morphology and to assure the simple and rapid reconstitution of nanoparticles from the electrospun product, the selection of suitable water-soluble polymers is crucial. Thus, we adopted our previously established formulation, which was based on P188 and PEO in the weight ratio of 1:1 [31]. P188, with its amphiphilic and surface-active properties, enables the rapid and successful reconstitution of nanoparticles from the electrospun product and improves the stability of nanoparticles in the reconstituted dispersion [51]; however, it does not enable the formation of nanofibers by the electrospinning process [52]. Thus, a well-spinnable hydrophilic polymer, namely PEO, was added to enable the formation of nanofibers. PEO was shown to improve the nanofiber contact with water and enable faster nanofiber dissolution [53,54]. Additionally, PEO in combination with sodium dodecyl sulfate has been already investigated for other similar purposes, such as embedding the multiwalled carbon nanotubes into polymer nanofibers [55].

The composition of the formulation has been adjusted for parenteral application, as SPIONs show great potential for their use in magnetic hyperthermia and as negative contrast agents in magnetic resonance imaging [3]. Based on the literature, data on the selected polymers, PEO and P188, are considered suitable for the parenteral application [51] and do not contribute importantly to the tonicity of the dispersion [56].

To define the composition of the prior-drying dispersion with the highest possible SPION content in the electrospun product, we performed the preliminary experiments in which we electrospun prior-drying SPION dispersions with different contents of SPIONs (Supplementary Materials Table S1), using the same process and ambient parameters as described in Section 2.4.2. The preliminary experiments with a 2.4.% (*w*/*v*) polymer concentration, namely PEO and P188 in a weight ratio of 1:1, in prior-drying SPION dispersions had shown that the highest possible SPION content in the electrospun product, which still enables a rapid, simple, and efficient reconstitution of SPIONs, is 65% (*w*/*w*) (Supplementary Materials Figure S2). The maximal content of SPIONs in the electrospun product achieved was higher compared to the silica-coated SPION clusters’ content, which was 50% (*w*/*w*) as published previously [31]. The results indicate that the investigated formulation based on the combination of hydrophilic polymers P188 and PEO has a different capacity for the incorporation of different types and sizes of nanoparticles. The size of the SPIONs in the present study was (53.5 ± 6.5) nm, which is much smaller compared to the silica-coated SPION clusters (200–350 nm), for which efficient incorporation was reported previously [31]. The surface characteristics of both types of nanoparticles were also different since, in the current study, we investigated the drying of charged citrate-coated SPIONs, whereas a previous investigation reported drying of SPION clusters coated with non-porous silica [31]. This indicates that the surface characteristics of nanoparticles can affect the maximal achievable nanoparticle content in the electrospun product.

The preliminary experiments with prior-drying SPION dispersion with 2.4% (*w*/*v*) polymers revealed that the SPION content affects the morphology of the electrospun product. The electrospun products were in the form of spheres, which were connected with thin fibers (Supplementary Materials Figure S3). According to the literature, such a product is formed when the polymer concentration is below the critical concentration, i.e., the concentration where smooth nanofibers are formed. At lower polymer concentrations, the surface tension and the applied electric field may cause the polymer chains to break into fragments before reaching the collector [57]. These polymer fragments cause the formation of beads or beaded nanofibers [36], which were observed in our preliminary experiments (Supplementary Materials Figure S3).

Based on the results of the preliminary experiments, we selected the final composition of the electrospun product to be the one with 65% (*w*/*w*) of SPIONs. Then, we investigated the influence of the polymer concentration on the drying of SPION dispersions. We compared the formulation from preliminary experiments, prepared from the prior-drying SPION dispersion with 2.4% (*w*/*v*) polymer concentration, with formulations prepared from prior-drying SPION dispersions with a 4.2% (*w*/*v*) and 6.4% (*w*/*v*) polymer concentration, to achieve the formation of the electrospun product in the form of smooth nanofibers, which would enable the easy, quick, and efficient reconstitution of SPIONs.

#### 3.2.2. Rheological Properties of Prior-Drying Samples

**Figure 2:**
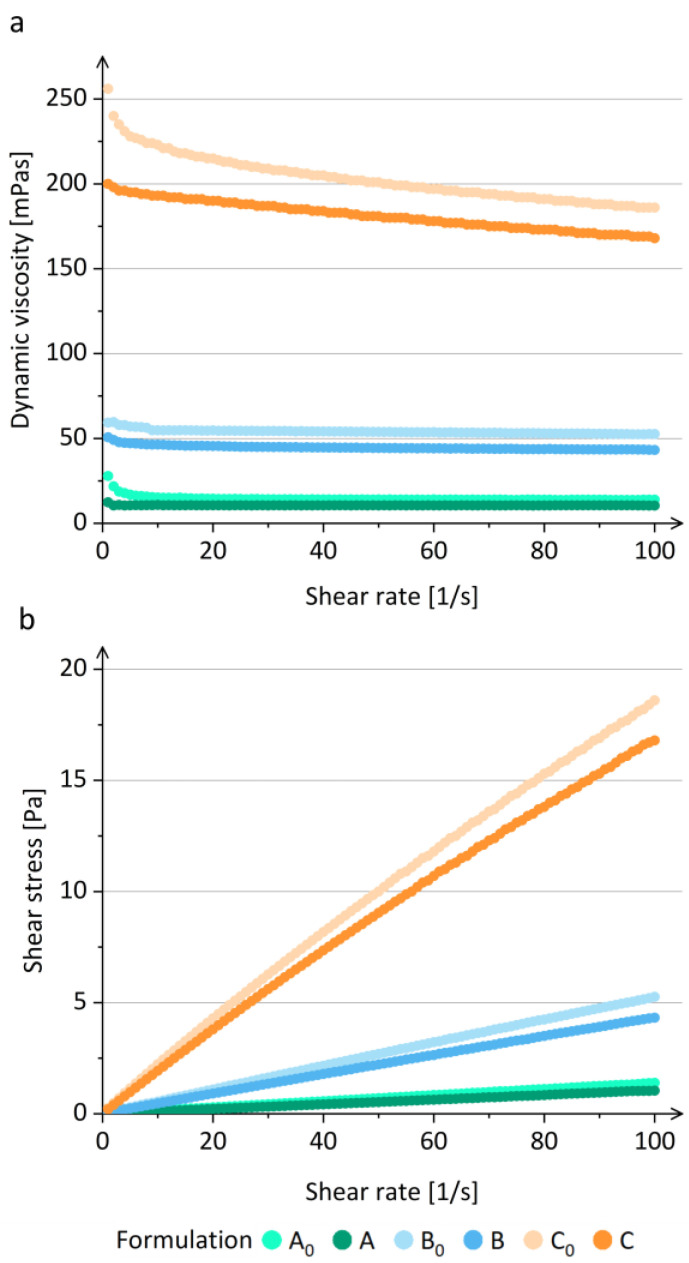
(**a**) Viscosity and (**b**) flow curves of prior-drying samples of formulations A_0_, A, B_0_, B, C_0_, and C.

Since the polymer concentration in the prior-drying dispersion influences the rheological properties of the prior-drying samples [36,38,58], and this affects the electrospinning process [35,36,37,38], we investigated the dynamic viscosity of all prior-drying samples. Prior-drying samples A and A_0_ with a 2.4% (*w*/*v*) polymer concentration and prior-drying samples B and B_0_ with a 4.2% (*w*/*v*) polymer concentration showed typical viscoelastic behavior of Newtonian fluids with a constant viscosity irrespective to an increase in the shear rate (1–100) s^−1^ (Figure 2a) [59]. The prior-drying samples C and C_0_ with a 6.4% (*w*/*v*) polymer concentration showed a decrease in viscosity with the increasing shear rate, indicating non-Newtonian (pseudoplastic) behavior (Figure 2a), which is typical for polymer solutions, as described in the literature [60,61]. During the shear process, the polymer molecules are orientated parallel to the direction of the shear, which results in elongation, which decreases the flow resistance and the bulk viscosity of polymer dispersions at higher shear rates [61].

The rheological measurements confirmed that the viscosity of polymer solutions is concentration dependent, as described in the literature [58,62]. It was shown that the viscosity increases with the increasing polymer concentration, while the addition of SPIONs in the polymer solution caused a significant decrease in viscosity (prior-drying samples A vs. A_0_, B vs. B_0_, and C vs. C_0_) (Figure 2a). The results showed that the SPIONs added to the polymer solution affect the prior-drying sample viscosity differently from silica-coated SPION clusters, which significantly increased the viscosity [31]. The difference observed might be due to the significantly smaller size of SPIONs ((53.5 ± 6.5) nm) compared to silica-coated SPION clusters (200–350 nm). Moreover, a similar behavior was also observed for the shear stress of the investigated prior-drying samples (Figure 2b).

In addition to the dynamic viscosity, the plastic and elastic modulus of prior-drying samples were also investigated. All samples demonstrated a higher plastic modulus compared to elastic modulus (Supplementary Materials Figure S4), which is a required rheological property for successful electrospinning [35]. Viscoelastic force and surface tension both tend to stabilize the polymer jet, which is elongated in the external electric field, enabling the evaporation of the solvent and the deposition of dry nanofibers onto the grounded collector [33]. Thus, it was confirmed that the investigated prior-drying samples had suitable viscoelastic properties for electrospinning.

#### 3.2.3. Conductivity of Prior-Drying Samples

Conductivity measurements revealed that the increase in polymer concentration affected the conductivity of prior-drying samples. The conductivity increased significantly with an increase in polymer concentration in prior-drying samples (Table 2). The increase in conductivity with increasing polymer concentration might be due to the impurities in the polymers used as reported previously [31]. The addition of SPIONs to the polymer solution led to a significant increase in the sample conductivity (Table 2; prior-drying samples A_0_ vs. A, B_0_ vs. B, and C_0_ vs. C). Since SPIONs are composed of conductive material, namely iron oxide [63], the effect on the sample conductivity after their addition was expected. The SPION concentration in prior-drying samples was increased proportionally to the polymer concentration, to keep the desired polymer-to-SPION weight ratio constant in all investigated formulations. The increase in conductivity when comparing the polymer solutions A_0_ and B_0_ or B_0_ and C_0_ is much smaller than the increase in conductivity comparing prior-drying SPION dispersions A and B or B and C (Table 2). Thus, it was shown that the SPION concentration has a bigger impact on the conductivity of prior-drying samples than polymer concentration.

The measured conductivities of polymer solutions were slightly different from the previously published data on similar aqueous PEO solutions. Uyar and Besenbacher (2009) determined the conductivity of a 3.0% (*w*/*v*) aqueous PEO solution at 25 °C to be approximately 77 µS/cm [64]. They also evaluated the conductivities of 3.5 and 4.0% (*w*/*v*) aqueous PEO solutions at 25 °C, which were similar to the conductivity of 3.0% (*w*/*v*) aqueous PEO solution [64]. Thus, it could be assumed that P188 contributes to the total conductivity of the blend solution with PEO. Compared to the prior-drying SPION dispersion C, the addition of SPIONs into the polymer solution C_0_ increased the conductivity more than the addition of silica-coated SPION clusters, which were previously shown to increase the conductivity of 6.4% (*w*/*v*) polymer solution to 459 µS/cm and enable the formation of nanofibers [31]. Since the addition of SPIONs has an important impact on the conductivity of polymer solution, they may also affect the spinnability of obtained dispersion. However, based on the literature data [31], the conductivities of our prior-drying SPION dispersions A, B, and C were suitable for electrospinning.

#### 3.2.4. Physical Stability of Prior-Drying SPION Dispersions

**Figure 3:**
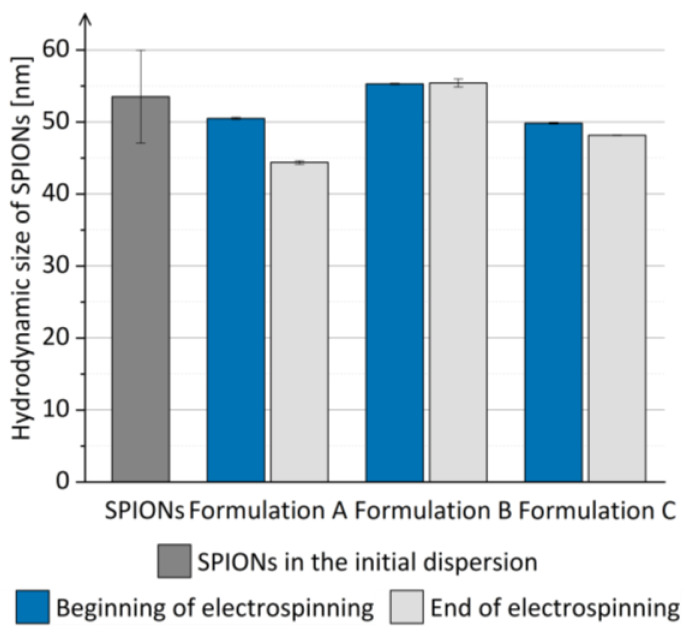
The mean hydrodynamic size of SPIONs in prior-drying SPION dispersions A, B, and C during the duration of electrospinning compared to the mean hydrodynamic size of SPIONs in the initial SPION dispersion.

The hydrodynamic size of SPIONs in the prior-drying SPION dispersions was comparable to the hydrodynamic size of SPIONs in the initial dispersion (Figure 3) and did not increase significantly when the prior-drying SPION dispersions were left undisturbed for 1 h. Thus, the aggregation of SPIONs in the prior-drying SPION dispersion, which might hinder the efficient SPION dispersion drying, was excluded, and the prior-drying SPION dispersions showed sufficient physical stability for at least 1 h, which equals the duration of the electrospinning process in the current study.

### 3.3. Evaluation of the Electrospun Products

#### 3.3.1. Visual Appearance of the Electrospun Products

**Figure 4:**
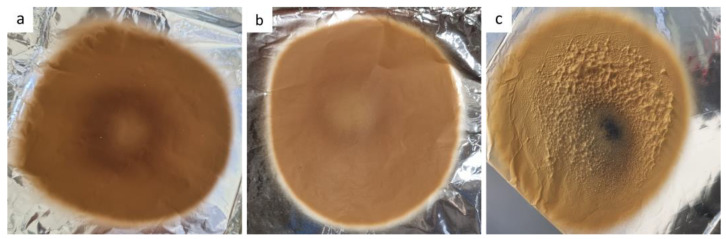
Product of electrospinning of (**a**) formulation A, (**b**) formulation B, and (**c**) formulation C collected on aluminum foil.

The electrospinning of SPION dispersions in polymer solutions enabled the formation of non-powdered products, which were deposited in a regular circular shape on the grounded collector (Figure 4). However, the electrospun products were not as homogenous as expected based on the literature data [31]. The electrospinning of prior-drying samples with different polymer and SPION concentrations resulted in electrospun products with a different visual appearance. The electrospun products A and B were colored light brown with a slightly darker circle in the center of the product and had a smooth surface (Figure 4a,b). The electrospun product C exhibited a rough surface with a dark brown-to-black colored center (Figure 4c). The dark circle in the middle of the electrospun product C might thus be a consequence of higher polymer and/or SPION concentration in prior-drying SPION dispersion C, which led to its significantly higher viscosity and conductivity when compared to the other two investigated prior-drying SPION dispersions (Figure 2 and Table 2). The dark-colored area in the middle of the electrospun product C might be due to (i) aggregation of SPIONs in the electric field during the electrospinning process or (ii) higher content of SPIONs in the dark central part of the electrospun product compared to the surrounding area. Therefore, we conducted additional investigations of this specific part of the electrospun product C, regarding the SPION content (TGA) and morphology of the product (SEM), which are presented in the following sections.

#### 3.3.2. Morphology of the Electrospun Products

**Figure 5:**
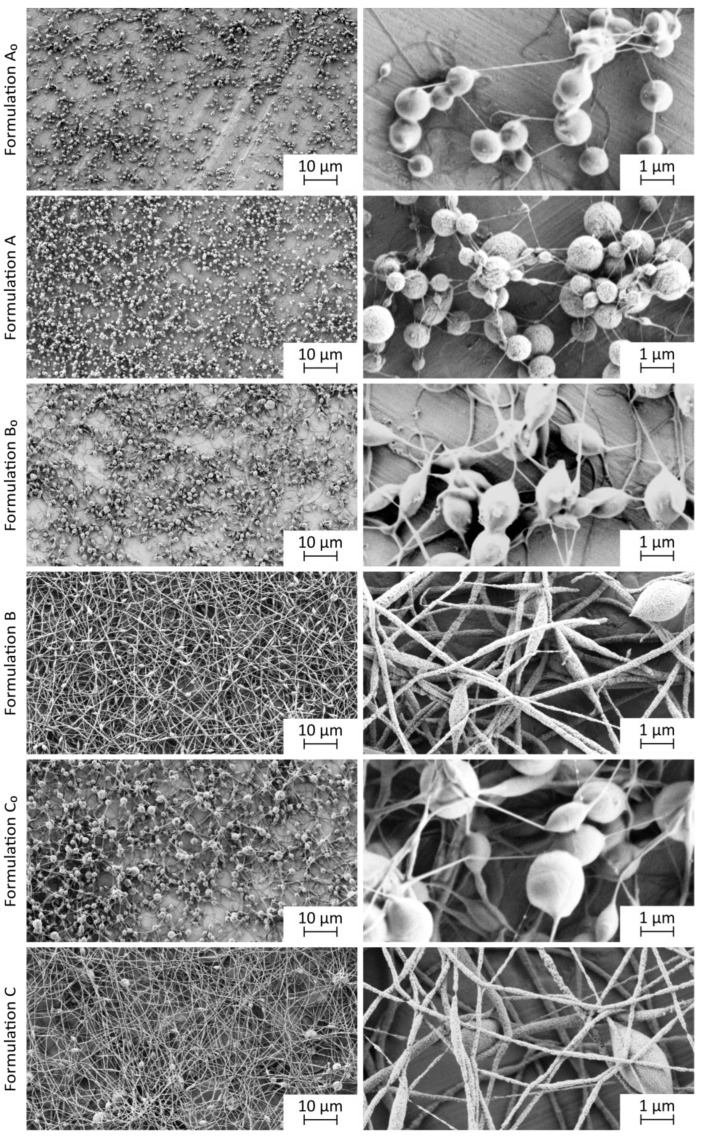
Representative SEM images of electrospun products at lower (**left**) and higher (**right**) magnification.

The SEM analyses of the electrospun products showed that the dispersion composition and its properties importantly affect the morphology of the electrospun products (Figure 5). The electrospun products A_0_, B_0_, and C_0_, which were prepared from the polymer solutions with significantly lower conductivities compared to prior-drying SPION dispersions A, B, and C, were in the form of spheres, interconnected with thin polymer fibers (Figure 5). According to the literature data, the low polymer concentration may lead to the formation of beaded fibers, and the electrospinning may turn into electrospraying below the critical polymer concentration [38]. However, the formation of the beads may also appear due to the capillary instability, as described in the literature [65]. The onset of capillary instability is normally prevented by the interactions of polymers in the dispersion and the electrical forces generated from the excess ions in the dispersion. The capillary instability during the electrospinning of polymer solutions of formulations A_0_, B_0_, and C_0_ occurred probably due to the reduced excess of the electrical charge. Thus, the capillary instability led to a collapse of the polymer solution jet into separated droplets, which solidified and formed beaded nanofibers [65].

SEM images show that higher polymer concentration in the prior-drying sample resulted in thicker fibers, connecting the polymer spheres (Figure 5, electrospun product C_0_). The morphology of the electrospun product C_0_ differed from the morphology of the electrospun product reported by Kajdič et al. (2018), who prepared smooth nanofibers from the prior-drying polymer solutions with the same composition by using a slower polymer solution flow rate (1.414 mL/h) [52]. However, not only polymer concentration but also viscosity, which was shown to be concentration dependent [38], might contribute to the non-fibrillar morphology of the electrospun products A_0_, B_0_, and C_0_. As described in the literature, the increase in viscosity might change the morphology of the electrospun product from beaded to smooth fibers [66]. However, our results do not show such a clear relationship between the polymer solution viscosity and electrospun product morphology. A small change in the shape of beads from spherical (electrospun product A_0_) to less spherical (electrospun product C_0_) was observed (Figure 5). Since the polymer solutions had a higher viscosity compared to corresponding prior-drying SPION dispersions (Figure 3) and since the electrospun products prepared from the prior-drying SPION dispersions B and C showed more fibrillar morphology than the electrospun products B_0_ and C_0_, which were prepared from pure polymer solutions (Figure 5), it was shown that conductivity also has an important influence on the morphology of the electrospun product.

**Figure 6:**
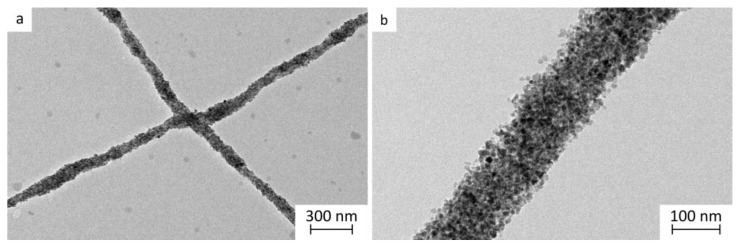
Representative TEM images of the electrospun product with 65% (*w*/*w*) of SPIONs at (**a**) lower and (**b**) higher magnification.

The SEM images revealed no SPIONs visible on the surface of electrospun products A, B, and C. Thus, we assumed that SPIONs might have been incorporated into the polymer matrix of nanofibers (Figure 5), and this was further confirmed with the TEM analysis (Figure 6). The electrospun product A was comparable to the electrospun products A_0_, B_0_, and C_0_. It was prepared from the prior-drying SPION dispersion with the lowest concentration, viscosity, and conductivity among investigated prior-drying SPION dispersions. It exhibited a spherical morphology with thin, presumably pure polymer fibrillar connections (Figure 5). Although the conductivity of prior-drying SPION dispersion A was much higher than the conductivity of prior-drying polymer solutions A_0_, B_0_, and C_0_ (Table 2), the viscosity of the prior-drying SPION dispersion A was the lowest among all investigated samples. The formation of nanofibers was enabled by the addition of SPIONs to the polymer solution, which per se resulted in a spherical electrospun product (polymer solutions B_0_ and C_0_; Figure 5). Based on the obtained results, it can be concluded that the conductivity and viscosity of prior-drying samples affected the morphology of the electrospun products. However, the formation of the beads may be related to the capillary instability, as reported previously [33,65].

As described, the addition of SPIONs led to significantly higher conductivity of prior-drying samples (Table 2) but slightly lower viscosity of prior-drying SPION dispersions B and C compared to polymer solutions B_0_ and C_0_. The nanofiber diameter of the electrospun product C ((225 ± 96) nm) was not significantly different from the nanofiber diameter of electrospun product B ((140 ± 76) nm (Supplementary Materials Figure S5). This is not in line with the literature data reporting that higher conductivity results in thinner nanofibers [35]. However, the produced nanofibers with SPIONs (65%, *w*/*w*) of formulation B had significantly smaller fiber diameters compared to the nanofibers with silica-coated SPION clusters (50%, *w*/*w*; (476 ± 199) nm), as reported previously [31]. Although the electrospun products of formulations B and C had a morphology of nanofibers, some beads were formed, which could be associated with the capillary instability of the prior-drying SPION dispersion jet [65].

The microscopical investigation of the morphology of the dark central part of the electrospun product C revealed that its morphology does not differ from the morphology of other parts of the electrospun product (Supplementary Materials Figure S6). Based on the morphology, stability of prior-drying SPION dispersion C, and hydrodynamic size of SPIONs after the reconstitution from the electrospun product C, which are described in the following sections, we concluded that SPION aggregation was not the reason for the dark appearance of the central part of the electrospun product C.

#### 3.3.3. SPION and Water Content in the Electrospun Products

The SPION content in the electrospun products was determined by the TGA, since polymers used in the formulations (PEO and P188) undergo complete thermal decomposition in the temperature range between 400 and 500 °C [67,68], whereas the SPIONs do not. The TGA of electrospun products revealed that the SPION contents in electrospun products (Table 3) were in good agreement with the theoretical SPION content (65% (*w*/*w*); Supplementary Materials Figure S7), indicating that SPIONs were successfully incorporated in the electrospun products, without any loss, regardless of the polymer concentration in prior-drying SPION dispersions. Since we performed the sampling of electrospun products from different locations on the collector and since the standard deviation of SPION content determined was <1.00%, we can conclude that SPIONs were homogeneously distributed in the electrospun products (Table 3).

TGA revealed that the electrospun products contained less than 0.74% (*w*/*w*) of water (Table 3), confirming electrospinning to be an efficient drying method, which is in concordance with the literature data. The water residual in electrospun products can be associated with water used as a dispersion medium in the prior-drying samples and with the hygroscopic properties of PEO and P188 [31,51].

The additional TGA of the dark central part of the electrospun formulation C revealed that it contained 66.56 ± 0.21% (*w*/*w*) of SPIONs and 0.21 ± 0.11% (*w*/*w*) of water. Thus, the analysis showed no difference in the SPION or water content between the darker central part and the rest of the sample. This indicates that color differences are not due to uneven SPION distribution in the electrospun product.

The magnetization measurements with VSM showed that the investigated SPIONs had superparamagnetic properties at room temperature, with the magnetization of 58.52 Am^2^/kg at 1 T (Supplementary Materials Figure S8), which is in line with our previous studies [69,70]. The magnetization of electrospun product B was 39.60 Am^2^/kg at 1 T (Supplementary Materials Figure S8). Thus, the SPION content in the electrospun product B, calculated based on the VSM data, was determined to be ~67.7% (*w*/*w*), which is in agreement with TGA results (66.51 ± 0.66%, *w*/*w*). Thus, it can be concluded that the SPIONs retained their magnetization during their transformation into the electrospun dry product as expected.

#### 3.3.4. FTIR Analysis of the Electrospun Products

**Figure 7:**
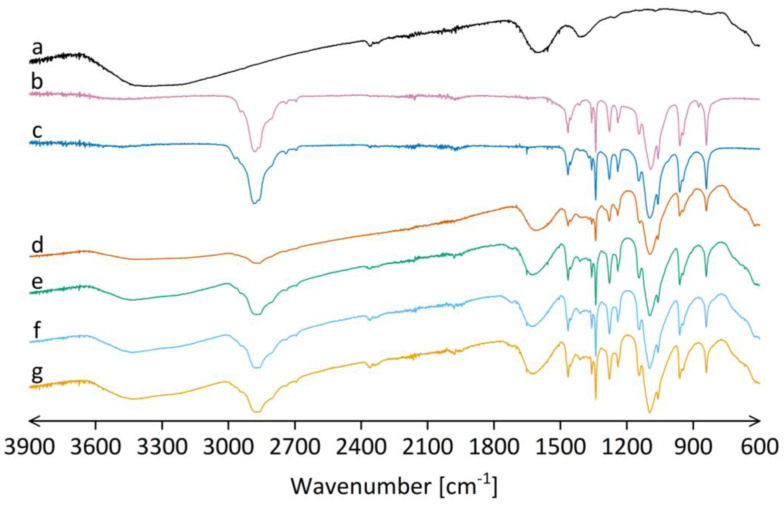
FTIR spectra of (**a**) oven-dried initial SPION dispersion, (**b**) PEO powder, (**c**) P188 powder, (**d**) physical mixture of PEO, P188, and oven-dried initial SPION dispersion in weight ratio comparable to electrospun products of formulations A, B, and C (17.5: 17.5: 65.0, respectively), and electrospun product of (**e**) formulation A, (**f**) formulation B, and (**g**) formulation C.

FTIR spectra of SPIONs, powdered polymers (PEO and P188), physical mixture of polymers and SPIONs, and the electrospun products A, B, and C revealed that there were no intermolecular interactions between the excipients and SPIONs in the electrospun products (Figure 7). All the characteristic peaks of SPIONs and polymers were preserved in the FTIR spectra of electrospun products without any shifts. Thus, the results indicate that the concentration of polymers in the prior-drying SPION dispersion does not have any effect on the possible interactions between the components in the electrospun products.

### 3.4. Reconstitution of SPIONs

The evaluation of the polymer impact on the hydrodynamic size and zeta potential of SPIONs in the polymer solution was performed before the reconstitution experiments. It revealed that the SPION hydrodynamic size was not affected by the presence of polymers in the dispersion medium, since the hydrodynamic size of SPIONs in the initial dispersion ((53.5 ± 6.5) nm) did not change significantly when SPIONs were added to the 0.035% (*w*/*v*) polymer solution ((52.3 ± 0.5) nm). On the contrary, the zeta potential of SPIONs ((−32.6 ± 7.7) mV) was significantly reduced in the presence of polymers ((−19.6 ± 4.7) mV). The decrease in zeta potential in the presence of polymers does not necessarily result in the lower physical stability of SPION dispersions, since polymers (especially P188) might improve the stability of the SPION dispersion by steric effect [51].

The reconstitution of SPIONs from electrospun products in purified water revealed that their reconstitution can be achieved in less than 3 min by simple vortex mixing, which is in accordance with the literature data on similar dry products [31].

**Figure 8:**
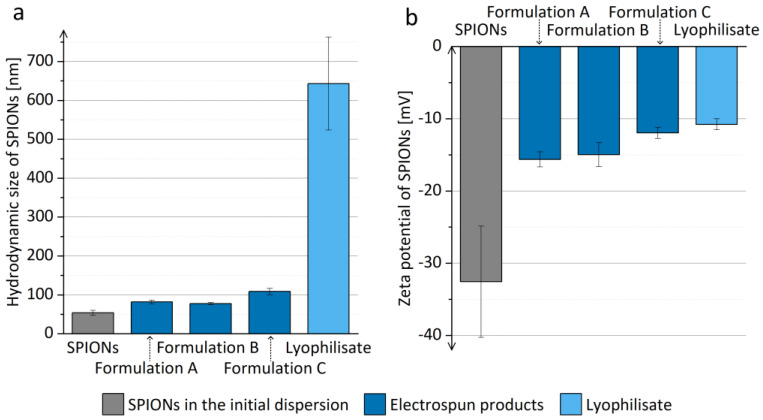
(**a**) Hydrodynamic size and (**b**) zeta potential of SPIONs in the initial dispersion and in the dispersions obtained by reconstitution of electrospun products of formulations A, B, and C, and lyophilizate in purified water.

The electrospun products represent a dry formulation of SPIONs, which can be reconstituted and thus transformed in liquid dispersion just prior to the parenteral application (e.g., for magnetic hyperthermia or as contrast agents for magnetic resonance imaging). The polymers in the electrospun product are not removed prior to application; thus, it is of great importance that they are safe for parenteral application in the used concentrations [51]. They may have an important impact on the SPION hydrodynamic size, as observed in the present study, where an increase in the hydrodynamic size of SPIONs after the reconstitution from the electrospun products compared to the hydrodynamic size of SPIONs in the initial dispersion was observed (Figure 8a). SPION hydrodynamic size was bigger than the hydrodynamic size of SPIONs in the initial dispersion, which could indicate the limited extent of SPION aggregation after the reconstitution. The reconstituted SPIONs dispersion with the presence of polymers was also analyzed with TEM, confirming the unchanged SPIONs’ morphology and nanocrystal size, as compared to the initial SPIONs’ dispersion (Supplementary Materials Figure S9). The increased SPIONs’ hydrodynamic size after the reconstitution can be assigned to the influence of polymers in the reconstituted dispersion, as SPIONs also showed a less negative zeta potential after their reconstitution from all electrospun products (Figure 8b). The reconstituted SPION dispersions had a pH of approximately 7.31, which is close to the ideal pH value for parenteral formulations (7.35–7.45) [71].

The different polymer concentrations and consequent viscosities and conductivities of prior-drying samples, which led to electrospun products with different morphologies, also affected the hydrodynamic size of SPIONs after their reconstitution. The mean hydrodynamic size of SPIONs after the reconstitution from the electrospun product C was significantly bigger compared to the mean hydrodynamic size of SPIONs after the reconstitution from the electrospun products A and B (Figure 8a). However, the hydrodynamic sizes of SPIONs after the reconstitution from the electrospun products A and B did not differ significantly irrespective of the differences in their morphology. Thus, we can conclude that the polymer concentration of prior-drying samples affects the hydrodynamic size of reconstituted SPIONs via its contribution to the higher conductivity of prior-drying dispersions. However, the contribution of the higher polymer concentration of prior-drying samples to the higher conductivity of prior-drying SPION dispersions is less important than the contribution of the added SPIONs (Table 2). Thus, it can be concluded that the dispersion parameter (besides polymer to SPION weight ratio), which affects the properties of the dry electrospun product and the efficiency of SPION reconstitution the most, is not the polymer concentration but rather the SPION concentration in prior-drying SPION dispersion, which was increased proportionally to the polymer concentration to keep the polymer-to-SPION weight ratio constant, resulting in dry products with 65% (*w*/*w*) SPION content. The difference in the morphology of the electrospun products A and B, which was presumably caused by the change in polymer concentration, had no important effect on the reconstitution efficiency of SPIONs from both electrospun products.

**Figure 9:**
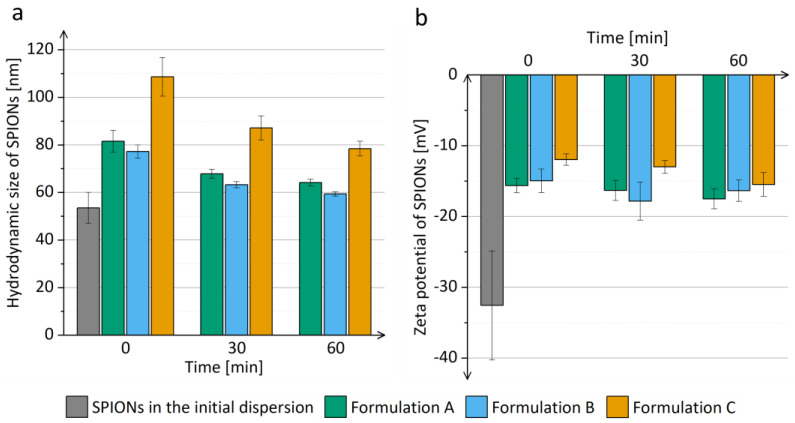
(**a**) Hydrodynamic size and (**b**) zeta potential of SPIONs in dispersions 1 h after the reconstitution from the electrospun products of formulations A, B, and C, as compared to SPIONs in the initial dispersion.

The SPION dispersions were stable up to 1 h after the reconstitution of SPIONs from the electrospun products, since the mean hydrodynamic size of SPIONs did not change significantly (Figure 9). A significantly smaller mean hydrodynamic size of SPIONs was observed 1 h after reconstitution compared to the size of SPIONs right after reconstitution (Figure 9a). Due to the tight packing of SPIONs in the polymer matrix during electrospinning, the polymer matrix did not dissolve completely during the reconstitution of SPIONs, but it dissolved later, when the sample was left standing at room temperature, which was observed as the mean SPION hydrodynamic size approaching the hydrodynamic size of SPIONs in the initial dispersion. However, further investigations are needed to explain this observation in more detail.

In order to thoroughly evaluate the electrospun product, we selected formulation B as the optimal formulation among the investigated ones. Thus, the prior-drying SPION dispersion with a 4.2% (*w*/*v*) polymer concentration was dried by electrospinning and by alternative drying methods, namely conventional drying in a heated oven and lyophilization. The reconstitution of the obtained dried products revealed electrospinning to be the best among the investigated drying methods since the obtained electrospun product was efficiently reconstituted. Contrarily, the reconstitution of SPIONs from the dried products, prepared by conventional drying in a heated oven (dry initial SPION dispersion and dry prior-drying SPION dispersion B), was not efficient. Reconstitution by vortex mixing was not successful; thus, an additional energy input, namely sonication, was used for reconstitution. The dry product was dispersed partially, but agglomerates were still visible to the naked eye, indicating the procedure to be inefficient. The main reason might be the exposure of the sample to 90 °C and the long duration of the drying process, as already described in the literature [25,31]. On the other hand, we managed to reconstitute SPIONs from the lyophilizate, but the SPION hydrodynamic size in the reconstituted dispersion was significantly bigger compared to the SPION hydrodynamic size after their reconstitution from the electrospun products (Figure 9a). The results confirmed that electrospinning is the most suitable method for drying SPION dispersions and their transformation into easily redispersible dry non-powdered product.

## 4. Conclusions

In conclusion, our study demonstrated the potential of electrospinning as a promising drying method for SPION dispersions. We successfully prepared a non-powdered dry product containing up to 65% (*w*/*w*) SPIONs, from which SPIONs can be easily and rapidly reconstituted. With the investigation of different polymer concentrations, namely a mixture of PEO and P188 in a weight ratio of 1:1, we showed that the polymer and SPION concentrations of the prior-drying samples affect the visual appearance and morphology of electrospun products. Our results revealed that a prior-drying SPION dispersion with the polymer concentration of 4.2% (*w*/*v*) leads to the formation of an easily dispersible electrospun product with fibrillar morphology and 65% (*w*/*w*) of SPIONs. To sum up, this investigation confirms the applicability of electrospinning as an efficient method for the transformation of SPION dispersions into a more user-friendly dry SPION formulation, which is ready for use after reconstitution.

## Figures and Tables

**Table 1:** Composition of the prior-drying samples (polymer solutions and SPION dispersions) and corresponding electrospun products.

	Prior-Drying Sample		Electrospun Product	
Formulation	Polymers (%, *w*/*v*)	SPIONs (%, *w*/*v*)	Polymers (%, *w*/*w*)	SPIONs (%, *w*/*w*)
A_0_	2.4	0.0	100	0
B_0_	4.2	0.0	100	0
C_0_	6.4	0.0	100	0
A	2.4	4.5	35	65
B	4.2	7.8	35	65
C	6.4	11.8	35	65

**Table 2:** Conductivities of polymer solutions (A_0_, B_0_, and C_0_) and prior-drying SPION dispersions (A, B, and C) at 25 °C.

Prior-Drying Sample	Conductivity at 25 °C (μS/cm)
A_0_	92.6 ± 0.8
B_0_	117.2 ± 0.5
C_0_	141.9 ± 1.0
A	422.5 ± 1.0
B	598.0 ± 0.0
C	999.2 ± 6.4

**Table 3:** SPION and water content in electrospun products, as determined by the TGA.

Electrospun Product	SPION Content (%, *w*/*w*)	Water Content (%, *w*/*w*)
A	66.11 ± 0.17	0.65 ± 0.08
B	66.51 ± 0.66	0.35 ± 0.04
C	66.80 ± 0.86	0.31 ± 0.02

## Data Availability

All research data needed to evaluate the conclusions in this paper are included in the paper and/or the Supplementary Materials. Additional data related to this paper can be obtained from the authors upon request.

## Supplementary Materials

The following supporting information can be downloaded at https://www.mdpi.com/article/10.3390/pharmaceutics15061619/s1, Figure S1: Schematic workflow of the overall work; Table S1: Composition of the prior-drying SPION dispersions (preliminary experiments); Figure S2: The hydrodynamic size of SPIONs after their reconstitution from the electrospun products compared to SPIONs in the initial dispersion (preliminary experiments); Figure S3: Representative SEM images of electrospun products (preliminary experiments) at lower (left) and higher (right) magnification; Figure S4: Plastic (G″) and elastic (G′) modulus of prior-drying samples of (a) formulation A_0_, (b) formulation A, (c) formulation B_0_, (d) formulation B, (e) formulation C_0_, and (f) formulation C; Figure S5: Histogram showing the nanofiber diameter distribution for formulations B, C, and C_0_; Figure S6: Representative SEM images of the dark central part of the electrospun product of formulation C at lower (left) and higher (right) magnification; Figure S7: Representative graph of the weight loss during the TGA of the electrospun product with 65% (*w*/*w*) SPION content; Figure S8: Representative graph of the magnetization as a function of the magnetic field for the initial SPIONs and electrospun products; Figure S9: Representative TEM images of reconstituted SPIONs at lower (left) and higher (right) magnification.
