# Supplementary material for: Influence of Polymer Concentration on Drying of SPION Dispersions by Electrospinning
Source: Pharmaceutics. 2023 May 30;15(6):1619. doi: 10.3390/pharmaceutics15061619 (PMC10302812; doi:10.3390/pharmaceutics15061619)
Supplement: Supplementary file 1 [file pharmaceutics-15-01619-s001.zip › pharmaceutics-2336965-supplementary.pdf]

pharmaceutics

Supplementary Materials

Influence of Polymer Concentration on Drying of SPION Dispersions by Electrospinning

Črt Dragar [1], Žan Rekar [1], Tanja Potrč [1], Sebastjan Nemec [1,2], Slavko Kralj [1,2,3] and Petra Kocbek [1,*]

[1] Department of Pharmaceutical Technology, Faculty of Pharmacy, University of Ljubljana, SI-1000 Ljubljana, Slovenia; crt.dragar@ffa.uni-lj.si (Č.D.); tanja.potrc@ffa.uni-lj.si (T.P.); sebastjan.nemec@ijs.si (S.N.); slavko.kralj@ijs.si (S.K.)
[2] Department for Materials Synthesis, Jožef Stefan Institute, SI-1000 Ljubljana, Slovenia
[3] Nanos SCI, Nanos Scientificae d.o.o, Ljubljana, SI-1000, Slovenia
* Correspondence: petra.kocbek@ffa.uni-lj.si

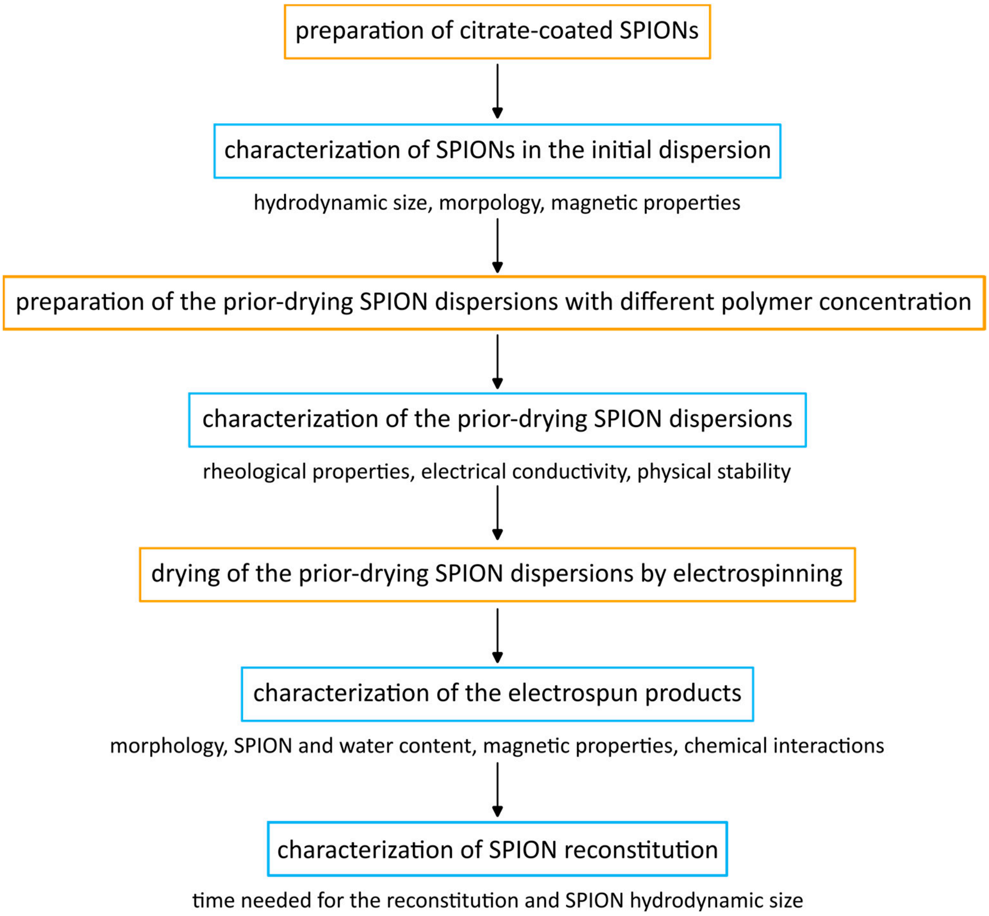

Figure S1. Schematic workflow of the overall work.

Table S1. Composition of the prior-drying SPION dispersions (preliminary experiments).

Formulation	w_{SPIONs} (in the product) [/]	SPIONs [mg]	PEO [mg]	P188 [mg]	Water [mL]
1	0.50	84	42	42	3.5
2	0.55	103	42	42	3.5
3	0.60	126	42	42	3.5
4	0.65	156	42	42	3.5
5	0.70	196	42	42	3.5
6	0.75	252	42	42	3.5
7	0.80	336	42	42	3.5

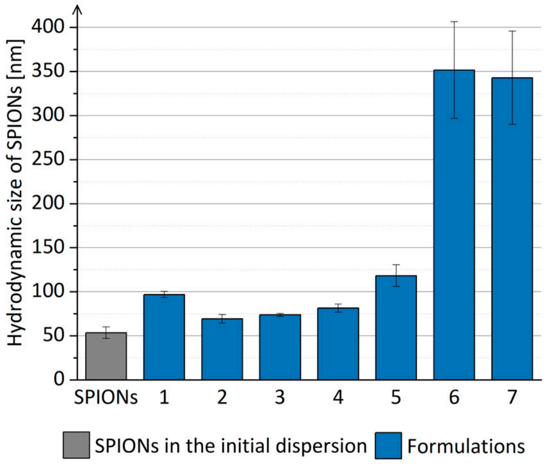

Figure S2. The hydrodynamic size of SPIONs after their reconstitution from the electrospun products compared to SPIONs in the initial dispersion (preliminary experiments).

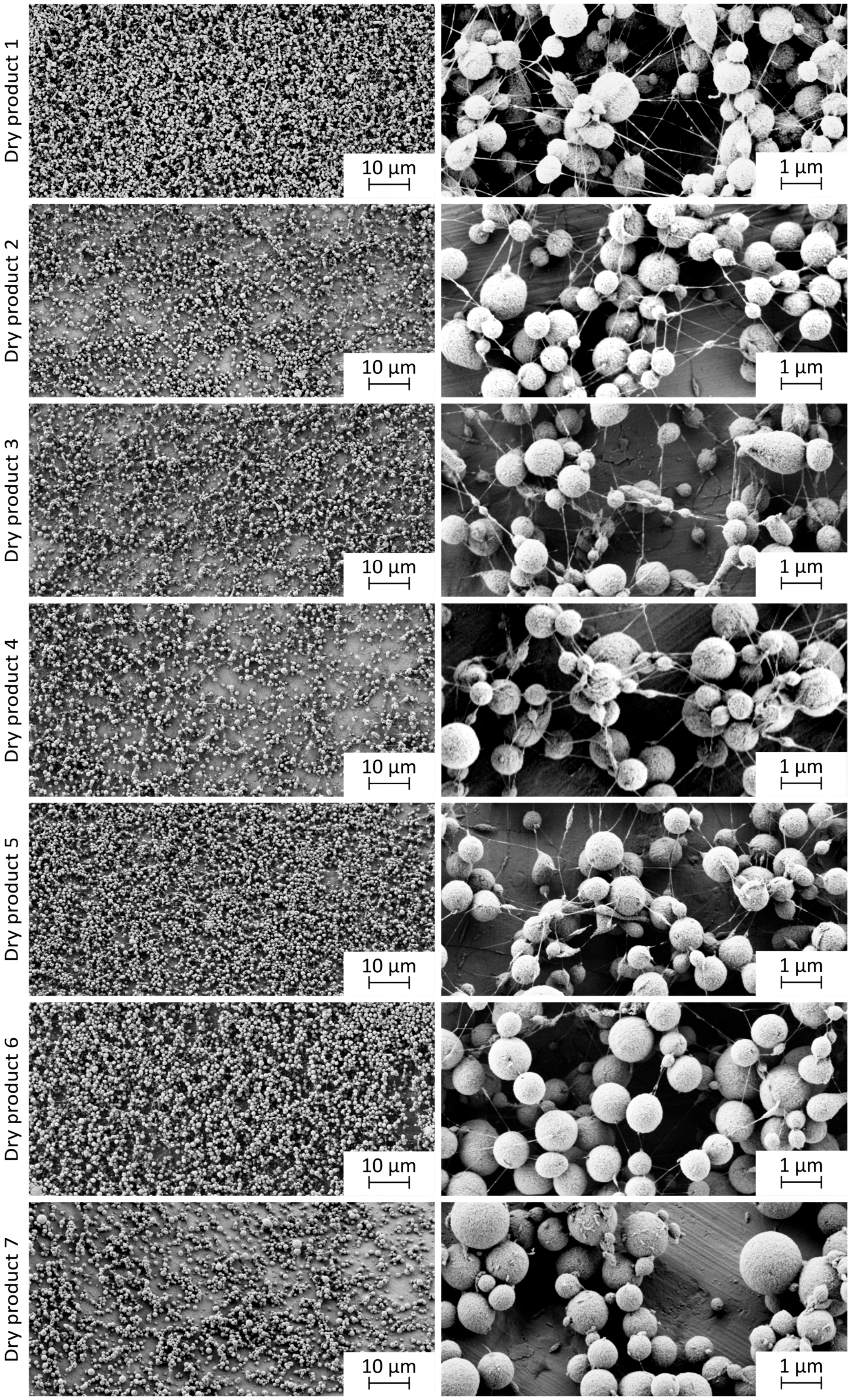

Figure S3. Representative SEM images of electrospun products (preliminary experiments) at lower (left) and higher (right) magnification.

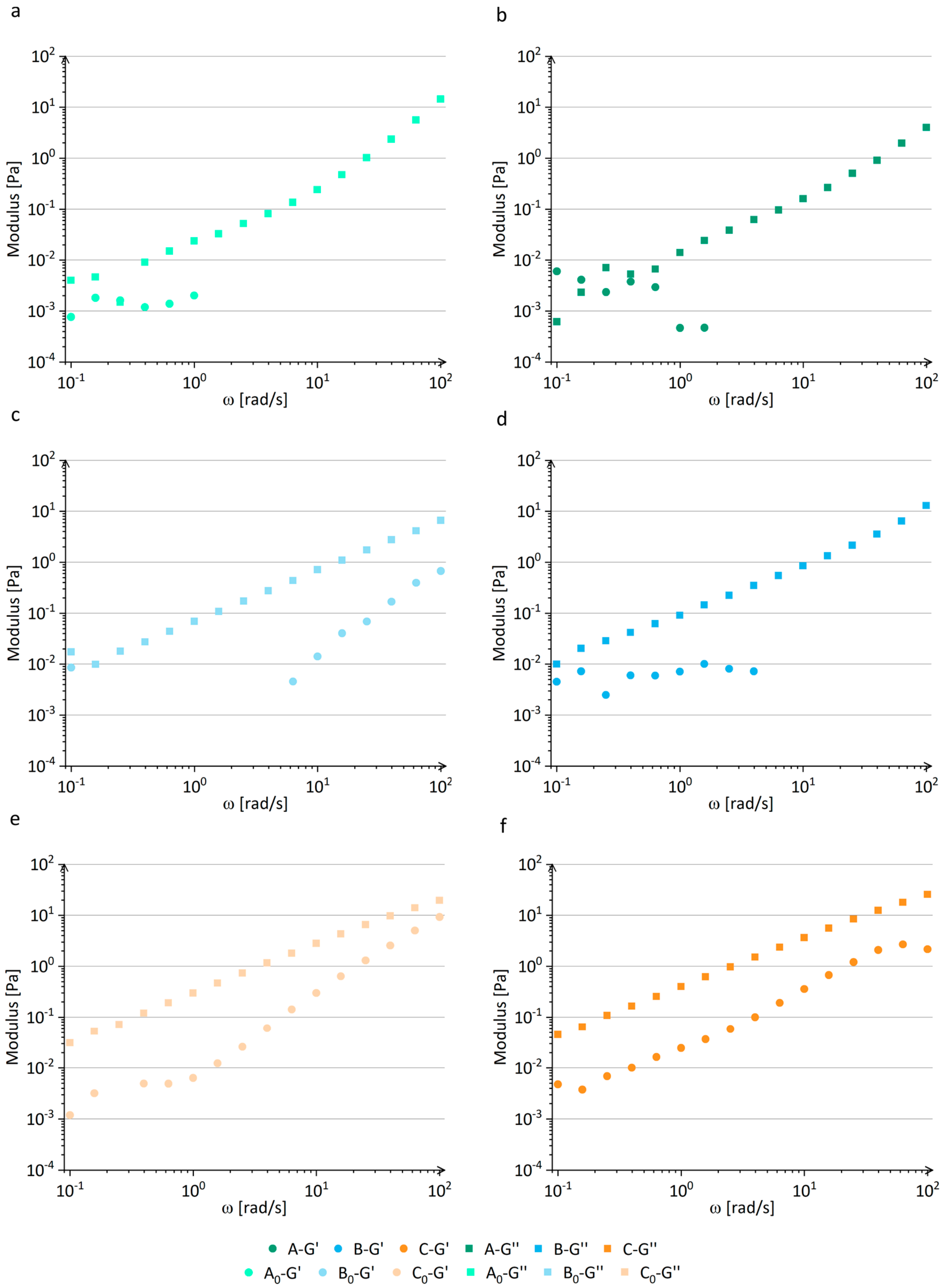

Figure S4. Plastic (G’’) and elastic (G’) modulus of prior-drying samples of (**a**) formulation A_0, (**b**) formulation A, (**c**) formulation B_0, (**d**) formulation B, (**e**) formulation C_0, and (**f**) formulation C.

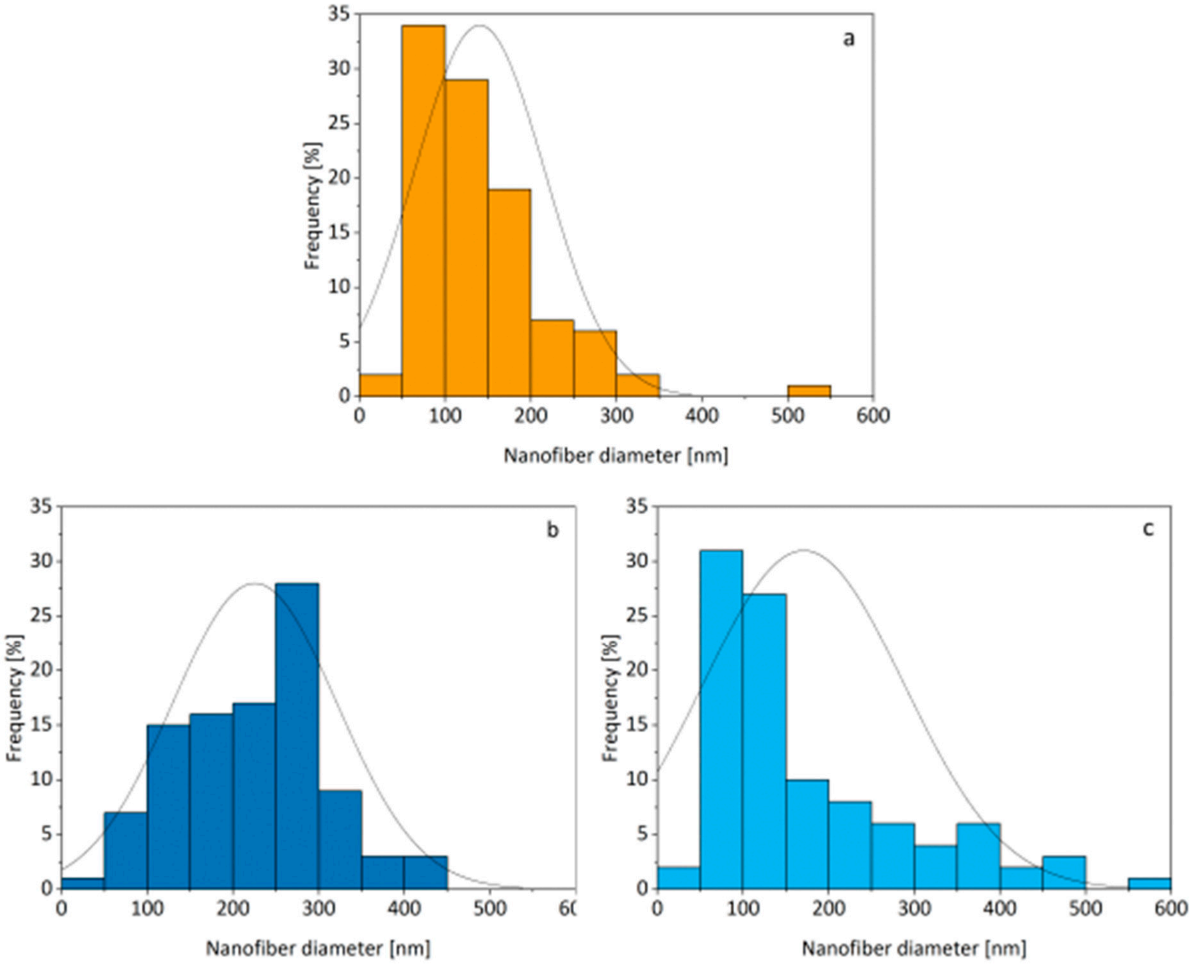

Figure S5. Histogram showing the nanofiber diameter distribution for (**a**) formulation B, (**b**) formulation C, and (**c**) formulation C_0.

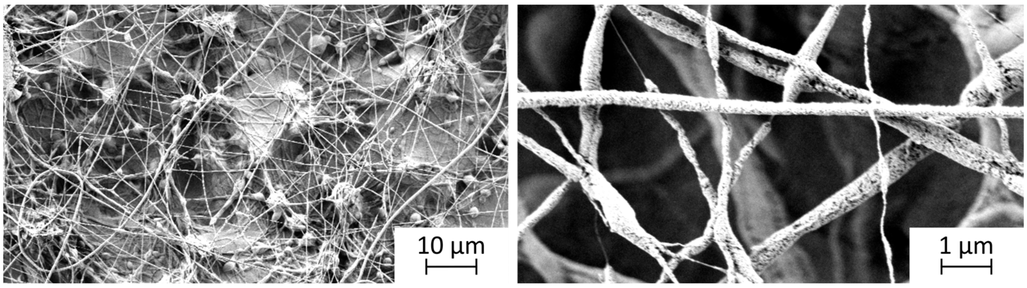

Figure S6. Representative SEM images of the dark central part of the electrospun product of formulation C at lower (left) and higher (right) magnification.

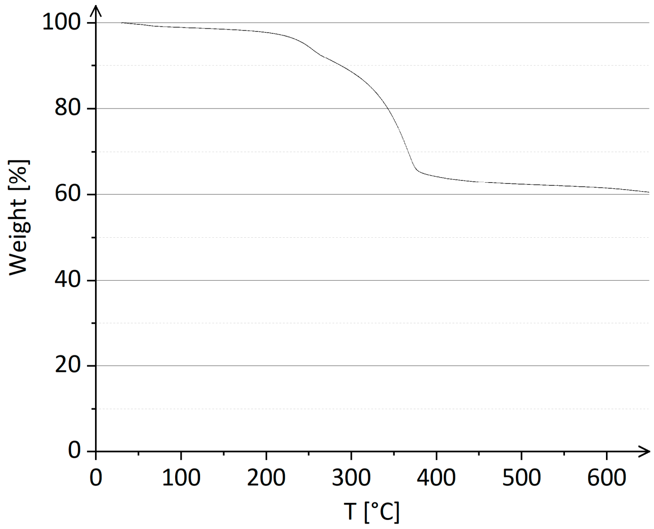

Figure S7. Representative graph of the weight loss during the TGA of the electrospun product with 65 % (w/w) SPION content.

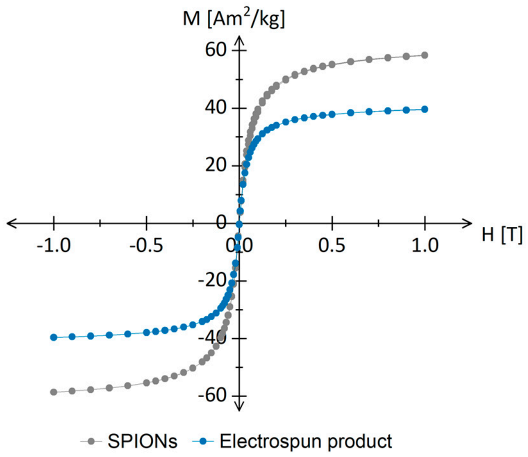

Figure S8. Representative graph of the magnetization (M) as a function of the magnetic field (H) for the initial SPIONs and electrospun product.

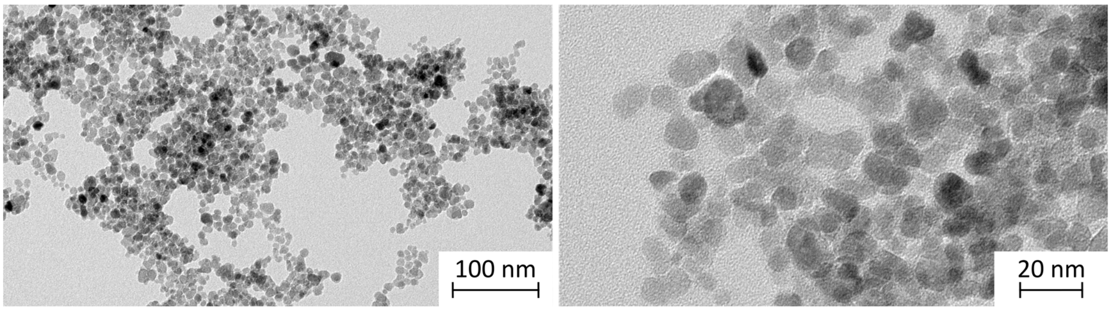

Figure S9. Representative TEM images of reconstituted SPIONs at lower (left) and higher (right) magnification.
